# Molecular Analysis of Tick-Borne Bacterial Pathogens from Ticks Infesting Animal Hosts in Kyrgyzstan, 2021

**DOI:** 10.3390/microorganisms12061046

**Published:** 2024-05-22

**Authors:** Yu Jung Kim, Ji Ye Seo, Jin Seo Park, Seong Yoon Kim, Bekbolsun Aknazarov, Nurzina Atabekova, Hee Il Lee

**Affiliations:** 1 Division of Vectors and Parasitic Diseases, Korea Disease Control and Prevention Agency (KDCA), 187 Osongsaenmyeong 2-ro, Osong-eup, Heungdeok-gu, Cheongju 28159, Republic of Korea; hoiyui@korea.kr (Y.J.K.); seojiye02@korea.kr (J.Y.S.); js722@korea.kr (J.S.P.); gunbo0402@korea.kr (S.Y.K.); 2 Faculty of Veterinary Medicine, Kyrgyz National Agrarian University Named after K. I. Skryabin, Bishkek 720005, Kyrgyzstan; aknazaroov-61@mail.ru (B.A.); atbn.7@mail.ru (N.A.)

**Keywords:** tick-borne pathogen, *Anaplasma*, *Ehrlichia*, tick, Kyrgyzstan

## Abstract

This study investigated the prevalence of *Anaplasma* and *Ehrlichia* in 494 engorged ticks collected from various animal hosts, including cattle, horses, sheep, chickens, dogs, and cats, in six regions of northern Kyrgyzstan. Ten tick species, belonging to two families and six genera, were identified based on *CO1*, 16S rRNA, and *ITS2* genes: *Argas persicus* (26.5%), *Haemaphysalis punctata* (18.0%), *Dermacentor* spp. (16.0%), *Rhipicephalus annulatus* (11.8%), *R*. *turanicus* (10.9%), *D*. *marginatus* (7.7%), *Hyalomma scupense* (4.5%), *Hy*. *marginatum* (3.8%), *R*. *sangineus* complex (0.6%), and *Ornithodoros lahorensis* (0.2%). PCR analysis revealed a 15.0% (74/494) overall infection rate of *Anaplasma* and *Ehrlichia*. *Anaplasma* species were found in six tick species and were identified as *A. bovis* (*n* = 44), *Anaplasma* spp. (*n* = 20), *A. ovis* (*n* = 5), and *A. capra* (*n* = 2). *Ehrlichia* species were found only in *H. punctata* (*n* = 5) and identified as *E. chaffeensis* (*n* = 1) and *Ehrlichia* spp. (*n* = 4). Additionally, two *H. punctata* were co-infected with *Anaplasma* and *Ehrlichia*. This is the first study to investigate tick-borne bacterial pathogens in ticks collected from animal hosts in Kyrgyzstan. Our findings contribute to a better understanding of the epidemiology and emergence of tick-borne infections in Kyrgyzstan.

## 1. Introduction

Kyrgyzstan is a landlocked country bordered by Kazakhstan, Tajikistan, Uzbekistan, and the Xinjiang Uygur Autonomous Region (XUAR, northwestern China). Over 85% of Kyrgyzstan’s agricultural land area comprises mountain pastures and hay fields, which are favorable for raising livestock [1,2]. As a traditional nomadic country, approximately 40% of the population of Kyrgyzstan is engaged in agriculture and animal husbandry, and the production of meat, milk, and wool by farming animals such as sheep, goats, and cows is economically vital [3,4].

Ticks are one of the major ectoparasitic arthropods affecting the health of domestic animals [5,6]. They may transmit a wide range of pathogens, including bacteria (*Anaplasma*, *Ehrlichia* [7], *Rickettsia* [8]), viruses (*Dabie bandavirus* [9] and *Orthoflavivirus encephalitidis* [10,11]), and protozoa (*Babesia* and *Theileria* [12,13]). When large numbers of ticks feed on animals, they can reduce milk and meat production, thereby decreasing the value of domestic animals. In addition, controlling and preventing ticks and tick-borne diseases are expensive, leading to significant financial losses [5,14]. The environmental conditions, such as climate change, affect tick populations and change their geographic distribution, which may lead to an increase in tick-borne diseases [15]. Therefore, the identification of vector ticks and tick-borne pathogens (TBPs) is crucial for the clinical diagnosis, treatment, and surveillance of tick-borne diseases.

Tick species are typically identified based on their morphological characteristics, which require well-trained technicians for accurate identification [16]. This process becomes more challenging when ticks are engorged with blood or are physically damaged [17]. To address this challenge, various molecular identification techniques have been employed using mitochondrial (cytochrome oxidase subunit 1; CO1 [18], 12S and 16S ribosomal RNA [19]) and nuclear markers (internal transcribed spacer 1 and 2, 18S, 28S [19,20]). In Kyrgyzstan, tick identification has been performed mainly through the microscopic examination of several ticks of the genera *Argas*, *Dermacentor*, *Haemaphysalis*, *Hyalomma*, *Ixodes*, and *Rhipicephalus* reported in Kyrgyzstan [21,22]. These ticks often act as vectors for various TBPs [5,23,24,25,26].

In addition, various TBPs have been detected in animal hosts, including cattle (*Anaplasma centrale*, *A. phagocytophilum* like-1, *A. capra*, *A. marginale*, *Babesia bovis*, *B. bigemina*, *B. major*, *Theileria orientalis*, and *T. annulata*) [22,27,28,29,30], sheep (*A. phagocytophilum*-like 1, *A. ovis*, and *A. capra*) [22,31], and dogs (*B. vogeli*, *B. vulpes*, and *T. ovis*) [22,32] in Kyrgyzstan. Although the molecular studies on tick-transmitted pathogens in the blood of several animals in Kyrgyzstan have been reported, no research has been conducted on tick-borne bacterial pathogens in tick-infesting animal hosts. Therefore, we aimed to investigate the distribution of tick species and detect *Anaplasma* and *Ehrlichia* species in ticks collected from northern Kyrgyzstan.

## 2. Materials & Methods

### 2.1. Study Area and Tick Collection

**Figure 1:**
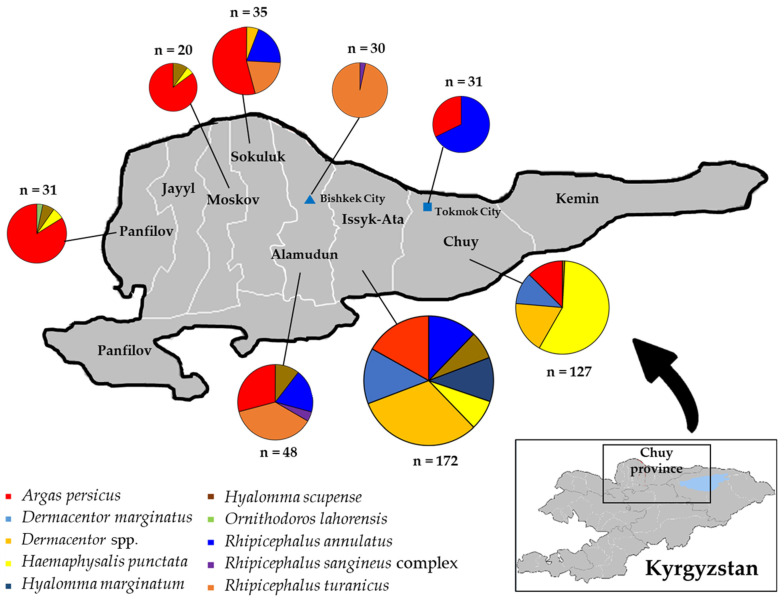
A map of tick species’ composition in the different sampling regions of Chuy Province, Kyrgyzstan, in 2022. The different colors indicate different tick species. The sizes of the circles represent the number of collected ticks and the pie charts illustrate their relative proportions in each collection region.

In 2021, 494 ticks were collected across six regions (Alamudun, Chuy, Issyk-Ata, Moskov, Sokuluk, and Panfilov) and two cities (Bishkek and Tokmok) in the Chuy Province in Kyrgyzstan (Figure 1). The Chuy province is located in the northern part of the country and is characterized by a humid continental climate. In the survey period between March and June in the collection area, the average temperature was 14.8 °C with a mean humidity of 63%. Partially or fully engorged ticks were collected from animal hosts such as cats, cattle, chickens, dogs, horses, and sheep. Ticks on cats, chickens, and dogs were mainly collected from animals living in residential houses and had not been given any specific anti-parasitic treatment. Additionally, ticks were collected from stray dogs that had been trapped, neutered, and sterilized at a veterinary clinic to control their population. Samples were randomly removed using tweezers from the entire surface of the animal hosts. The collected ticks were preserved in 70% ethanol, and information such as the location, host preference, number of ticks, and date of collection was recorded and transported to the laboratory for species identification and pathogen detection. Individual ticks were placed in 2.0 mL cryovials and then frozen at −80 °C until DNA extraction.

### 2.2. DNA Extraction

Genomic DNA was extracted using the Clear-S™ Quick DNA extraction kit (INVIRUSTECH, Gwangju, Republic of Korea) for each tick. Briefly, 600 µL of lysis buffer and 2.8 mm ceramic (zirconium oxide) beads were used to homogenize the ticks using a Precellys 24 tissue homogenizer (30 Hz for 2 min) (Bertin Instruments, Montigny-le-Bretonux, France). After centrifuging the tick lysate at 12,000× *g* for 10 min, the supernatant was used for DNA isolation, according to the manufacturer’s instructions. Isolated DNA was eluted in 50 µL of elution buffer and kept at −80 °C for subsequent analysis.

### 2.3. Molecular Identification of Tick Species and Tick-Borne Pathogens

The molecular identification of tick species was performed by amplifying the sequences of cytochrome oxidase subunit 1 (*CO1*), 16S rRNA, and the internal transcribed spacer 2 (*ITS2*) genes [18,33,34]. For the screening of *Anaplasma* and *Ehrlichia* using the 16S rRNA gene, the AE1-F/AE1-R primer set was first used for amplification, and then PCR products were used as templates for the nested PCR of *Anaplasma* spp. and *Ehrlichia* spp. using the EE3/EE4 and HE1/HE3 primer sets, respectively [35,36,37]. Conventional and nested PCRs were performed using an AccuPower^®^ PCR PreMix (Bioneer, Seoul, Republic of Korea). For the primary PCR, the reaction mix consisted of 1 µL (10 pmol) of each primer, 13 µL of ddH_2_O, and 5 µL of total DNA extracted from the ticks in a total volume of 20 µL. For the nested PCR, 1 µL of the corresponding primary products was used as templates. All the reactions were carried out in a C1000 Touch Thermal Cycler (Bio-Rad Laboratories, Hercules, CA, USA), using the previously published primers and protocols listed in Table 1. The total genomic DNA of laboratory strains of *A. phagocytophilum* and *E. chaffeensis* provided by the Division of Bacterial Diseases and the Division of Zoonotic and Vector Borne Disease Research KDCA served as positive controls. The amplified products were visualized via capillary electrophoresis using an automated QIAxcel system (QIAGEN, Hilden, Germany).

### 2.4. Nucleotide Sequencing and Phylogenetic Analysis

All positive PCR products were sequenced using each primer set at BIOFACT (Daejeon, Republic of Korea). The generated sequences were compared to previously published sequences from the National Center for Biotechnology Information (NCBI, Bethesda, MD, USA). For phylogenetic analysis, the nucleotide sequences were aligned with each homologous sequence using CLUSTAL Omega (v. 1.2.1). Neighbor-joining phylogenetic trees were constructed using the Kimura 2-parameter distance model in MEGA 5.2 software. To assess the bootstrap values of the obtained tree, 1000 bootstrap replicates were performed.

### 2.5. Statistical Analysis

A 95% confidence interval (CI) for all estimates was calculated using https://www.medcalc.org/calc/rate_ci.php (MedCalc Software Ltd. Version 22.018; accessed on 23 January 2024).

## 3. Result

### 3.1. Molecular Identification of Tick Species

**Figure 2:**
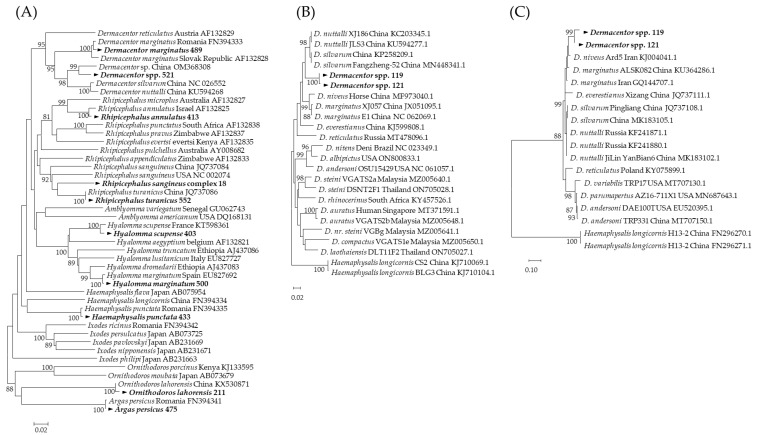
(**A**) Phylogenetic analysis based on *CO1* (597 bp), (**B**) 16S rRNA genes (345 bp), and (**C**) *ITS2* (655 bp). The sequences identified in this study are indicated in bold (▶). The phylogenetic tree was constructed using the neighbor-joining method (**A**) and maximum likelihood method (**B**,**C**) based on the Kimura 2-parameter mode. The numbers on the branches indicate bootstrap percentages based on 1000 replications. The cut-off value for the consensus tree was 60%. Over 60% of the total number were collected in Issyk-Ata (*n* = 172, 34.8%) and Chuy (*n* = 127, 25.7%) followed by Alamudun (*n* = 48, 9.7%), Sokuluk (*n* = 35, 7.1%), Panfilov (*n* = 31, 6.3%), Tokmok City (*n* = 31, 6.3%), Bishkek City (*n* = 30, 6.1%), and Moskov (*n* = 20, 4.0%) (Figure 1). Regarding tick infestation in host animals, 494 of partially or fully engorged ticks were collected from sheep (*n* = 161, 32.6%), followed by cattle (*n* = 122, 24.7%), chickens (*n* = 104, 21.1%), horses (*n* = 71, 14.4%), dogs (*n* = 34, 6.9%), and cats (*n* = 2, 0.4%) (Supplementary Table S1).

In northern Chuy Province, Kyrgyzstan, 494 ticks were collected and molecularly identified. Among these 494 ticks, 10 species belonging to six genera were represented. The most dominant tick species was *Argas persicus* (*n* = 131, 26.5%), followed by *Haemaphysalis punctata* (*n* = 89, 18.0%), *Dermacentor* spp. (*n* = 79, 16.0%), *Rhipicephalus annulatus* (*n* = 58, 11.8%), *R. turanicus* (*n* = 54, 10.9%), *D*. *marginatus* (*n* = 38, 7.7%), *Hyalomma scupense* (*n* = 22, 4.5%), *Hy. marginatum* (*n* = 19, 3.8%), *R. sangineus* complex (*n* = 3, 0.6%), and *Ornithodoros lahorensis* (*n* = 1, 0.2%) (Figure 1, Supplementary Table S1). The *CO1* sequencing results confirmed that each tick sequence clustered at the species level (Figure 2A). However, the *R. sangineus* complex sequence identified in this study was placed in an independent cluster and was not grouped with other *Rhipicephalus* species sequences (Figure 2A), and some *Dermacentor* spp. were placed in different clusters with related reference sequences in the 16S rRNA (Figure 2B) and *ITS2* (Figure 2C) analyses.

### 3.2. Detection of Tick-Borne Pathogens in Ticks

A total of 494 tick specimens were screened for the presence of *Anaplasma* and *Ehrlichia* using the 16S rRNA gene. Seventy-one *Anaplasma* were identified, of which the most common species were *A. bovis* (*n* = 44, 8.9%; 95% CI: 0.06–0.12) followed by *Anaplasma* spp. (*n* = 20, 4.0%, 95% CI: 0.02–0.06), *A. ovis* (*n* = 5, 1.0%; 95% CI: 0.10–0.17), and *A. capra* (*n* = 2, 0.4%; 95% CI: 0.00–0.01) (Table 2). In the case of *Ehrlichia*, only five *H. punctata* collected from cattle and horses were infected with *E. chaffeensis* (*n* = 1, 0.2%; 95% CI: 0.00–0.02) and *Ehrlichia* spp. (*n* = 4, 0.8%; 95% CI: 0.00–0.02). In addition, ticks positive for *Ehrlichia* spp. and *E. chaffeensis* were coinfected with *A. capra* and *A. bovis*, respectively (Table 2).

*H. punctata* (*n* = 40, 44.9%) was the most prevalent tick species infected with *Anaplasma* and *Ehrlichia*, followed by *A. persicus* (*n* = 19, 14.5%), *R. annulatus* (*n* = 7, 12.1%), *R. turanicus* (*n* = 6, 11.1%), *Demacentor* spp. (2.5%, *n* = 2), *Hy. marginatum* (*n* = 1, 5.3%), and *H. scupense* (*n* = 1, 4.5%) (Table 2). According to the host, the ticks detached from sheep (*n* = 28, 17.4%) had a high tick-borne pathogen detection rate, followed by those from cattle (*n* = 22, 18.0%), horses (*n* = 14, 19.7%), chickens (*n* = 10, 9.6%), and dogs (*n* = 5, 5.9%). No pathogens were detected in any ticks taken from cats (Table 2). Ticks collected from the Chuy region (*n* = 34, 26.8%) had the highest positivity rate of *Anaplasma* and *Ehrlichia* followed by Issyk-Ata (*n* = 19, 11.0%), Sokuluk (*n* = 6, 17.1%), Bishkek City (*n* = 4, 13.3%), Moskov (*n* = 4, 20.0%), Panfilov (*n* = 3, 9.7%), Tokmok City (*n* = 3, 9.7%), and Alamudun (*n* = 3, 6.3%) (Supplementary Table S2).

The representative sequences of different hosts for *Anaplasma* and *Ehrlichia* species obtained in this study were deposited in the NCBI database with the following accession numbers: *A. bovis* (OR234597–OR23463), *A. capra* (OR150332), *A. ovis* (OR150327–OR150329), *Anaplasma* spp. (OR150334, OR150337, OR150343, OR150349), *E. chaffeensis* (OR140774), and *Ehrlichia* spp. (OR140773, OR140775–OR140777).

### 3.3. Molecular and Phylogenetic Analysis of Tick-Borne Pathogens

**Figure 3:**
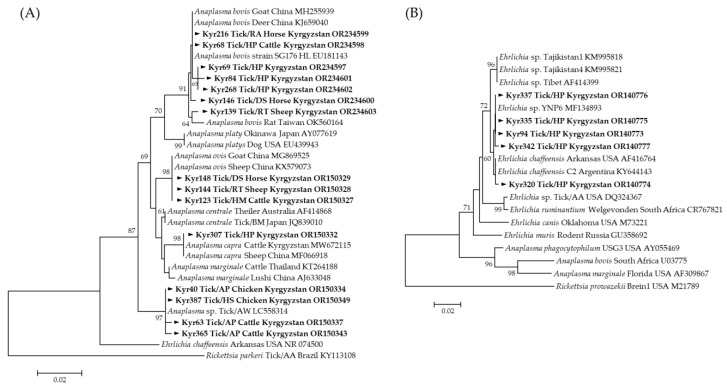
Phylogenetic relationships for *Anaplasma* and *Ehrlichia* based on the partial nucleotide sequence of (**A**) *Anaplasma* 16S rRNA, (**B**) *Ehrlichia* 16S rRNA. The neighbor-joining method was used to construct a phylogenetic tree. The numbers at the nodes represent the proportion of bootstrap values for the branch point. The positive sequences for *Anaplasma* and *Ehrlichia* identified in this study are indicated in bold (▶). Scale bars indicate sequence distances. Reference strains of *Anaplasma* and *Ehrlichia* with the host, country of detection, and the National Center for Biotechnology Information accession numbers are also shown. The cut-off value for the consensus tree was 60%. (AP: *A. persicus*, DS: *Dermacentor* spp., HM: *H. marginatum*, HP: *H. punctata*, RA: *R. annulatus*, RT: *R. turanicus*).

A total of 12 representative sequences for *Anaplasma* and 4 representative sequences for *Ehrlichia* were selected without duplicate sequences among those identified as positive for 71 *Anaplasma* and 5 *Ehrlichia* species, respectively (Table 2). Each pathogen was detected in two to three tick species. *A. bovis* sequences obtained from *H. punctata* and *R. annulatus* (OR234598) showed a 100% identity with *A. bovis* isolated from goats in China (MH255939). *A. bovis* sequences obtained from *R. turanicus* (OR234603) were similar to *A. bovis* isolated from rats in Taiwan (OK560164), and *A. bovis* sequences obtained from *Demacentor* spp. and *H. punctata* (OR234597 and OR23460-OR234602) revealed a 99.30–99.88% similarity with *A. bovis* isolated *from H. longicornis* in ROK (EU181143). *A. ovis* sequences obtained from *Hy. marginatum*, *R. turanicus*, and *Dermacentor* spp. (OR150327) exhibited a 100% identity with *A. ovis* detected in goats and sheep in China (MG869525 and KX579073, respectively.) *A. capra* sequences obtained from *H. punctata* (OR150332) shared 100% and 99.88% identities with *A. capra* isolated from cattle in Kyrgyzstan (MW672115) and sheep in China (MF066918), respectively. *Anaplasma* spp. sequences obtained from *A. persicus* and *H. scupense* (OR150334, OR150337, OR150343, OR150349) shared a 99.19–99.54% identity with that of *Anaplasma* spp. isolated from the soft tick, *A. walkerae*, in Zambia (LC558323). Phylogenetic analysis based on partial 16S rRNA sequences revealed that *A. bovis*, *A. capra*, *A. ovis*, and *Anaplasma* spp. detected in this study were included in the *Anaplasma* species (Figure 3A).

The results of the BLAST analysis showed that one positive sequence (OR140774) isolated from *H. punctata* revealed a 99.74% identity with *E. chaffeensis* obtained from the Arkansas strain in the USA (AF416764) and C2 strain in Argentina (KY644143). *Ehrlichia* spp. (OR140773–OR140777) revealed a 99.74–100% identity with *Ehrlichia* spp., which was deposited in GenBank (MF134893). Phylogenetic analyses demonstrated that the 16S rRNA sequences of *E. chaffeensis* and *Ehrlichia* spp. clustered with the previously documented sequences (Figure 3B).

## 4. Discussion

The present study investigated engorged ticks collected from animal hosts in six regions of Kyrgyzstan to identify their species and the prevalence of *Anaplasma* and *Ehrlichia* pathogens within them using molecular detection and phylogenetic analysis.

In accordance with previous studies, Fedorova et al. [21] and Aknazarov et al. [22] identified several tick species in Kyrgyzstan using morphological classification keys and confirmed *Ixodes persulcatus*, *Haemaphysalis punctata*, *H. erinacei*, *Hyalomma anatolicum*, *Hy. Scupense*, *Hy. Marginatum*, *Dermacentor marginatus*, *Rhipicephalus pumilio*, *R. sanguineus*, and *R. turanicus.* In our investigation, tick species were identified via molecular analysis, and *Rhipicephalus annulatus* and *Ornithodoros lahorensis* were newly identified.

The *R. sangineus* complex comprises several tick species, ranging from *R. sanguineus* to *R. turanicus* [38,39]. Owing to their close relationships and similar phenotypic features, the *R. sanguineus* complex is frequently misidentified as other species within the complex [40]. The taxonomic status of this group remains uncertain, with different classifications provided by various authors [41,42,43]. Although scanning electron microscopy (SEM) and transmission electron microscopy (TEM) have enabled the morphological distinction of *Rhipicephalus* species, genetic analysis remains the most reliable method for identifying the different species within the *R. sanguineus* complex [18,38,44]. In this study, three *Rhipicephalus* species sequences were placed between *R. sanguineus* and *R. turanicus* for phylogenetic analysis using *CO1* and were determined as the *R. sangineus* complex.

For tick species’ identification, *CO1* should be considered first; if *CO1* does not provide reliable results, 16S rRNA, *ITS2*, or 12S rRNA can be used [19]. Our study found that the sequences of *Dermacentor* spp. formed a clade distinct from other validated *Dermacentor* species in the phylogenetic analysis using *CO1*. Additional phylogenetic analysis was conducted using 16S rRNA and *ITS2* for cases in which *CO1* was not analyzable.

Little research has been conducted on the prevalence of tick-borne pathogens among ticks infesting animal hosts in Kyrgyzstan. Sang et al. [45] detected five species of piroplasms, *Babesia occultans*, *B. caballi*, *Theileria ovis*, *T. annulata*, and *T. equi*, in ticks collected from domestic animals in Kazakhstan. In Tajikistan, *Ehrlichia* spp. (4/382 pools, 1.1%) and *Theileria* spp. (3/92 pools, 3.3%) were detected in *Hyalomma anatolicum* detached from domestic animals [46]. Several studies have investigated the bacterial TBPs harbored by animal hosts in Kyrgyzstan. In 2019, Aktas [27] performed the first molecular epidemiological study on bovine piroplasmosis, including *B. major*, *T. orientalis*, and *T. annulata*, using cattle blood samples. Ozubek [28] identified the genotypes of *T. orientalis* in the blood of cattle using Single-Strand Conformation Polymorphism (SSCP) analysis. In 2022, *Anaplasma* species (*A. phagocytophilum* like-1, *A. ovis* and *A. capra*) were detected in cattle [29] and sheep [31] in nine regions of Kyrgyzstan. Recently, Aknazarov [22] reported that blood harvested from sick animals in Kyrgyzstan carried six pathogens, including *Anaplasma*, *Ehrlichia*, *Babesia*, *Theileria*, *Nuttalia*, and *Hemobartonella*, using Giemsa staining in 2023.

Although several studies of tick-borne pathogens have been reported in animal hosts in Kyrgyzstan, no information is available on using molecular tools to characterize their prevalence in ticks. The only pathogen identified in ticks is the tick-borne encephalitis virus (TBEV), which causes severe neurological disorders and is found in *Ixodes persulcatus* in the northern region [10,11]. Our investigation revealed that seven of the ten tick species including *H. punctata*, *A. persicus*, *R. annulatus*, *R. turanicus*, *Demacentor* spp., *Hy. marginatum*, and *H. scupense* were PCR-positive for four *Anaplasma* species (*A. bovis*, *A. capra*, *A. ovis*, and *Anaplasma* spp.) and two *Ehrlichia* species (*E. chaffeensis* and *Ehrlichia* spp.). In the literature, *Anaplasma* and *Ehrlichia* have been confirmed in *O. lahorensis* [47], *D. marginatus* [47], and *R. sangineus* complex [48], but no positive ticks were found in this study.

*Anaplasma* and *Ehrlichia* species are known as important tick-borne pathogens of both medical and veterinary importance [49]. These pathogens have been documented in numerous geographical areas and various animal species around the world [23,24,25,26]. According to a previous study, *A. bovis* was identified in *H. punctata* from birds in Spain [50], *A. ovis* was found in *R. turanicus*, and *R. annulatus* was identified in goats in Tunisia [51]. Recently, a new *Anaplasma* species, *A. capra*, was reported in goats in China [52]. *A. capra*, which was detected in two *H. punctata* specimens in our study, showed a 100% identity (43% coverage) with an *A. capra* sequence isolated from cattle in Kyrgyzstan [29]. In the case of *Ehrlichia* species, *E. chaffeensis* was detected in *Amblyomma parvum* ticks in Argentina [53], and *Ehrlichia* spp. was detected in *Hy. anatolicum*, Tajikistan [46].

Aknazarov et al. [22] revealed through the blood smear examination of sick animals in Kyrgyzstan that six species infesting sick animals were carriers of six pathogens (anaplasmosis, ehrlichiosis, babesiosis, theileriosis, nuttaliosis, and hemobartonellosis) during an investigation in 2021 and 2022. Our study revealed that ticks collected from the animals investigated in 2021 were infected with four species of *Anaplasma* and two species of *Ehrlichia*. Since pathogens and diseases were identified in both studies (ticks and animals), we believe that the collected species serve as vectors for these two tick-borne diseases and that anaplasmosis and ehrlichiosis silently circulate within this territory in Kyrgyzstan. This study was conducted only on partially or fully engorged ticks collected from animal hosts. Therefore, a limitation of this study is the possibility that the identified TBPs may have originated from animal hosts rather than vectors. Nevertheless, blood-feeding vectors are known to be reliable indicators of the presence or absence of pathogens in an area [54]. Our results also found co-infection with two species of pathogens (*E. chaffeensis* with *A. bovis*, and *Ehrlichia* spp. with *A. capra*) from two *H. punctata* specimens, and further investigation is expected to discover more co-infections with various pathogens, such as *Babesia* and *Theileria* [27,30,32]. To our knowledge, this is the first study to investigate tick-borne bacterial pathogens in the ticks collected from animal hosts in Kyrgyzstan.

Most populations in Kyrgyzstan depend on livestock for their livelihood. Owing to their nomadic lifestyle and geographical distribution, diverse animal and human exchanges with neighboring countries may facilitate the spread of ticks and tick-borne diseases. The current study focused mainly on the characterization of TBPs in tick vectors in a few areas of northern Kyrgyzstan. Therefore, further studies for various tick species and hosts should be continuously investigated across several regions to monitor the prevalence of TBPs. This study contributes to a better understanding of the epidemiology of tick-borne infections and the possibility of tick-borne diseases in Kyrgyzstan.

## Figures and Tables

**Table 1:** Primer sets used for the identification of tick and *Anaplasma* and *Ehrlichia* species.

Target	Primers	Sequence (5′ to 3′)		Amplicon Length (bp)	References
*CO*1	LCO1490 HC02198		GGTCAACAAATCATAAAGATATTGG TAAACTTCAGGGTGACCAAAAAATCA	710	[18]
16S rRNA	16S_F 16S_R2		TTAAATTGCTGTRGTATT CAACATCGAGGTCGCAAWCYA	395	[33]
ITS2	F3/1 R1/1		GGGTCGATGAAGAACGCAGCCAGC TTCAGGGGGTTGTCTCGCCTGATG	~1039	[34]
*Anaplasma* 16S rRNA	AE1-F AE1-R	1st PCR	AAGCTTAACACATGCAAGTCGAA AGTCACTGACCCAACCTTAAATG	1406	[35]
	EE3 EE4	2nd PCR	GTCGAACGGATTATTCTTTATAGCTTGC CCCTTCCGTTAAGAAGGATCTAATCTCC	926	
*Ehrlichia* 16S rRNA	AE1-F AE1-R	1st PCR	AAGCTTAACACATGCAAGTCGAA AGTCACTGACCCAACCTTAAATG	1406	[36]
	HE1 HE3	2nd PCR	CAATTGCTTATAACCTTTTGGTTATAAAT TATAGGTACCGTCATTATCTTCCCTAT	390	[37]

**Table 2:** Infection rate of *Anaplasma-* and *Ehrlichia*-positive tick species collected from animal hosts in Kyrgyzstan in 2021.

Host	Tick Species	Number of Tested Ticks	Detected Pathogens in Ticks						
			*A. bovis*	*A. capra*	*A. ovis*	*Anaplasma* spp.	*E. chaffeensis*	*Ehrlichia* spp.	Total (%)
Cat	*Rhipicephalus turanicus*	2	0	0	0	0	0	0	0
	Subtotal	2	0	0	0	0	0	0	0 (0)
Cattle	*Argas persicus*	41	0	0	0	9	0	0	9
	*Dermacentor marginatus*	28	0	0	0	0	0	0	0
	*Dermacentor* spp.	12	0	0	0	0	0	0	0
	*Haemaphysalis punctata*	21	10 (1 *)	0	0	0	1 (1 ^†^)	1	12
	*Hyalomma marginatum*	9	0	0	1	0	0	0	1
	*Hyalomma scupense*	2	0	0	0	0	0	0	0
	*Rhipicephalus annulatus*	9	0	0	0	0	0	0	0
	Subtotal	122	10	0	1	9	1	1	22 (18.0)
Chicken	*Argas persicus*	82	0	0	0	9	0	0	9
	*Haemaphysalis punctata*	2	0	0	0	0	0	0	0
	*Hyalomma scupense*	19	0	0	0	1	0	0	1
	*Ornithodoros lahorensis*	1	0	0	0	0	0	0	0
	Subtotal	104	0	0	0	10	0	0	10 (9.6)
Dog	*Rhipicephalus annulatus*	4	0	0	0	0	0	0	0
	*Rhipicephalus sangineus* complex	1	0	0	0	0	0	0	0
	*Rhipicephalus turanicus*	29	2	0	0	0	0	0	2
	Subtotal	34	2	0	0	0	0	0	2 (5.9)
Horse	*Dermacentor* spp.	6	1	0	1	0	0	0	2
	*Haemaphysalis punctata*	20	1	1 (1 **)	0	0	0	3 (1 ^††^)	5
	*Rhipicephalus annulatus*	45	7	0	0	0	0	0	7
	Subtotal	71	9	1	1	0	0	3	14 (19.7)
Sheep	*Argas persicus*	8	0	0	0	1	0	0	1
	*Dermacentor marginatus*	10	0	0	0	0	0	0	0
	*Dermacentor* spp.	61	0	0	0	0	0	0	0
	*Haemaphysalis punctata*	46	22	1	0	0	0	0	23
	*Hyalomma marginatum*	10	0	0	0	0	0	0	0
	*Hyalomma scupense*	1	0	0	0	0	0	0	0
	*Rhipicephalus sangineus* complex	2	0	0	0	0	0	0	0
	*Rhipicephalus turanicus*	23	1	0	3	0	0	0	4
	Subtotal	161	23	1	3	1	0	0	28 (17.4)
Total (%)	*Argas persicus*	131	0	0	0	19	0	0	19 (14.5)
	*Dermacentor marginatus*	38	0	0	0	0	0	0	0 (0.0)
	*Dermacentor* spp.	79	1	0	1	0	0	0	2 (2.5)
	*Haemaphysalis punctata*	89	33	2	0	0	1	4	40 (44.9)
	*Hyalomma marginatum*	19	0	0	1	0	0	0	1 (5.3)
	*Hyalomma scupense*	22	0	0	0	1	0	0	1 (4.5)
	*Ornithodoros lahorensis*	1	0	0	0	0	0	0	0 (0.0)
	*Rhipicephalus annulatus*	58	7	0	0	0	0	0	7 (12.1)
	*Rhipicephalus sangineus* complex	3	0	0	0	0	0	0	0 (0.0)
	*Rhipicephalus turanicus*	54	3	0	3	0	0	0	6 (11.1)
Total (%)		494	44 (8.9) (CI 0.06–0.12)	2 (0.4) (CI 0.0–0.01)	5 (1.0) (CI 0.10–0.17)	20 (4.0) (CI 0.02–0.06)	1 (0.2) (CI 0.00–0.01)	4 (0.8) (CI 0.00–0.02)	76 (15.3) (CI 0.12–0.19)

* co-infection with *E. chaffeensis*; ** co-infection with *Ehrlichia* spp.; ^†^ co-infection with *A. bovis.* ^††^ co-infection with *A. capra.*

## Data Availability

Data supporting the conclusions of this article are included within the article. The newly generated sequences were submitted to the GenBank database under the accession numbers OR150327–OR150329, OR150332, OR150334, OR150337, OR150343, OR150349, OR140773–OR140777, and OR234597–OR23463. The datasets used and/or analyzed during the present study are available from the corresponding author upon reasonable request.

## Supplementary Materials

The following supporting information can be downloaded at: https://www.mdpi.com/article/10.3390/microorganisms12061046/s1, Table S1: Geographical distribution of tick species collected from animal hosts in Kyrgyzstan in 2021; Table S2: Infection rates of *Anaplasma* and *Ehrlichia* in Kyrgyzstan by region.
