# Supplementary material for: Molecular Analysis of Tick-Borne Bacterial Pathogens from Ticks Infesting Animal Hosts in Kyrgyzstan, 2021
Source: Microorganisms. 2024 May 22;12(6):1046. doi: 10.3390/microorganisms12061046 (PMC11205634; doi:10.3390/microorganisms12061046)
Supplement: Supplementary file 1 [file microorganisms-12-01046-s001.zip › microorganisms-3005616-supplementary.pdf]

Supplementary Table S1. Geographical distribution of tick species collected from livestock in Kyrgyzstan in 2021.

Region	Host	Number of collected ticks										Total (%)
		Argas persicus	*Dermacentor marginatus*	*Dermacentor sp.*	*Haemaphysalis punctata*	*Hyalomma marginatum*	*Hyalomma scupense*	*Ornithodoros lahorensis*	*Rhipicephalus annulatus*	*Rhipicephalus sangineus* complex	*Rhipicephalus turanicus*	
Alamudun	Cattle	0	0	0	0	0	0	0	9	0	0	9
	Sheep	0	0	0	0	0	0	0	0	1	9	10
	Dog	0	0	0	0	0	0	0	0	1	9	10
	Chicken	14	0	0	0	0	5	0	0	0	0	19
	Subtotal	14	0	0	0	0	5	0	9	2	18	48 (9.7)
Bishkek City	Sheep	0	0	0	0	0	0	0	0	1	14	15
	Dog	0	0	0	0	0	0	0	0	0	15	15
	Subtotal	0	0	0	0	0	0	0	0	1	29	30 (6.1)
Chuy	Cattle	0	10	7	18	0	0	0	0	0	0	35
	Sheep	0	4	12	35	0	1	0	0	0	0	52
	Chicken	16	0	0	0	0	0	0	0	0	0	16
	Horse	0	0	4	20	0	0	0	0	0	0	24
	Subtotal	16	14	23	73	0	1	0	0	0	0	127 (25.7)
Issyk-Ata	Cattle	14	18	5	2	9	0	0	0	0	0	48
	Sheep	8	6	49	11	10	0	0	0	0	0	84
	Chicken	7	0	0	0	0	12	0	0	0	0	19
	Horse	0	0	0	0	0	0	0	21	0	0	21
	Subtotal	29	24	54	13	19	12	0	21	0	0	172 (34.8)
Moskov	Cattle	17	0	0	1	0	2	0	0	0	0	20
	Subtotal	17	0	0	1	0	2	0	0	0	0	20 (4.0)
Panfilov	Cattle	10	0	0	0	0	0	0	0	0	0	10
	Chicken	16	0	0	2	0	2	1	0	0	0	21
	Subtotal	26	0	0	2	0	2	1	0	0	0	31 (6.3)
Sokuluk	Dog	0	0	0	0	0	0	0	4	0	5	9
	Chicken	19	0	0	0	0	0	0	0	0	0	19
	Horse	0	0	2	0	0	0	0	3	0	0	5
	Cat	0	0	0	0	0	0	0	0	0	2	2
	Subtotal	19	0	2	0	0	0	0	7	0	7	35 (7.1)
Tokmok City	Chicken	10	0	0	0	0	0	0	0	0	0	10
	Horse	0	0	0	0	0	0	0	21	0	0	21
	Subtotal	10	0	0	0	0	0	0	21	0	0	31 (6.3)

Total (%)	Cattle	41	28	12	21	9	2	0	9	0	0	122 (24.7)
	Sheep	8	10	61	46	10	1	0	0	2	23	161 (32.6)
	Dog	0	0	0	0	0	0	0	4	1	29	34 (6.9)
	Chicken	82	0	0	2	0	19	1	0	0	0	104 (21.1)
	Horse	0	0	6	20	0	0	0	45	0	0	71 (14.4)
	Cat	0	0	0	0	0	0	0	0	0	2	2 (0.4)
	Total (%)	131 (26.5)	38 (7.7)	79 (16.0)	89 (18.0)	19 (3.8)	22 (4.5)	1 (0.2)	58 (11.7)	3 (0.6)	54 (10.9)	494(100)

Supplementary Table S2. Infection rates of *Anaplasma* and *Ehrlichia* in Kyrgyzstan by region.

Region	Number of tested ticks	Detected pathogens in ticks		Total (%)
		Anaplasma	*Ehrlichia*	
Alamudun	48	3		3 (6.3)
Bishkek City	30	4		4 (13.3)
Chuy	127	29	5	34 (26.8)
Issyk-Ata	172	19		19 (11.0)
Moskov	20	4		4 (20.0)
Panfilov	31	3		3 (9.7)
Sokuluk	35	6		6 (17.1)
Tokmok City	31	3		3 (9.7)
Total (%)	494	71	5	76 (15.4)
